# Detection of Metabolic Changes Induced via Drug Treatments in Live Cancer Cells and Tissue Using Raman Imaging Microscopy

**DOI:** 10.3390/bios9010005

**Published:** 2018-12-28

**Authors:** Mioara Larion, Tyrone Dowdy, Victor Ruiz-Rodado, Matthew W. Meyer, Hua Song, Wei Zhang, Dionne Davis, Mark R. Gilbert, Adrian Lita

**Affiliations:** 1 Neuro-Oncology Branch, Center for Cancer Research, National Cancer Institute, National Institutes of Health, Bethesda, MD 20892, USA; mioara.larion@nih.gov (M.L.); tyrone.dowdy@nih.gov (T.D.); victor.ruizrodado@nih.gov (V.R.-R.); hua.song@nih.gov (H.S.); zhangwe@mail.nih.gov (W.Z.); ddavis913@icloud.com (D.D.); mark.gilbert@nih.gov (M.R.G.); 2 ThermoFisher Scientific, 5225 Verona Road, Madison, WI 53711, USA; matt.meyer@thermofisher.com

**Keywords:** fibrosarcoma IDH1, NAD^+^ synthesis, Raman spectrometry, microscopy, single cell imaging, tissue imaging

## Abstract

Isocitrate dehydrogenase 1 (IDH1) mutations in gliomas, fibrosarcoma, and other cancers leads to a novel metabolite, D-2-hydroxyglutarate, which is proposed to cause tumorigenesis. The production of this metabolite also causes vulnerabilities in cellular metabolism, such as lowering NADPH levels. To exploit this vulnerability, we treated glioma and fibrosarcoma cells that harbor an IDH1 mutation with an inhibitor of nicotinamide adenine dinucleotide (NAD^+^) salvage pathway, FK866, and observed decreased viability in these cells. To understand the mechanism of action by which the inhibitor FK866 works, we used Raman imaging microscopy and identified that proteins and lipids are decreased upon treatment with the drug. Raman imaging showed a different distribution of lipids throughout the cell in the presence of the drug compared with the untreated cells. We employed nuclear magnetic resonance NMR spectroscopy and mass spectrometry to identify the classes of lipids altered. Our combined analyses point to a decrease in cell division due to loss of lipid content that contributes to membrane formation in the in vitro setting. However, the FK866 drug did not have the same potency in vivo. The use of Raman imaging microscopy indicated an opposite trend of lipid distribution in the tissue collected from treated versus untreated mice when compared with the cells. These results demonstrate the role of Raman imaging microscopy to identify and quantify metabolic changes in cancer cells and tissue.

## 1. Introduction

Raman spectroscopy has been used for decades to determine vibrational models linked with structural changes of small molecules [1]. Due to recent progress in diode lasers and Rayleigh suppression filters and low noise charged coupled devices (CCDs), the study of biological systems became possible in the form of Raman imaging microscopy [2,3]. Focusing the laser through the microscope allows the collection of Raman spectra for each pixel and subsequent movement of the stage permits the acquisition of spectra in a new position. This approach provides a chemical and biochemical mapping of the entire cell in the culture media, for example, when a water immersion objective is used. Therefore, the technique is non-destructive, non-invasive, and can accept a wide range of samples from bulk to microscopic, from solids to liquids or gasses, and in the absence of any external label. The advances in Raman imaging microscopy, together with its broad applicability in the biological fields, has prompted the emergence of Ramanomics as one of the “omics” fields [4]. The advantages of using Raman are multiple, one of which includes that this technique has the potential to report on metabolic changes in absence of any tag. In the recent years, Raman imaging microscopy has emerged as a new optical technique to map metabolic changes altered at the level of organelles, but also on larger scale such as tissues, with the potential to decipher the cancer biology one organelle at a time, to discover biomarkers, to delineate the border between the normal and cancer cells, and provide prognostic and diagnostic insights [5,6,7,8,9]. In addition, the Raman imaging microscopy approaches could map the location of different drugs in tissue, assuming that it is sensitive enough and that the signal coming from the drug does not overlap significantly with the biological signal. Therefore, Raman imaging microscopy could serve as a detection method for the exogenous drug mapping and has the potential to add spatial and temporal distribution of these drugs.

The majority of lower grade glioma possess an activating mutation in a metabolic gene called isocitrate dehydrogenase I (IDH1). The IDH1 mutant (IDH1*^mut^*) enzyme produces a novel metabolite D-2-hydroxyglutarate (D-2HG), which is proposed to contribute to tumorigenesis via alteration of the epigenome [10,11,12,13,14]. These alterations lead to global changes in gene expression and block the cellular differentiation [12]. However, very little is known about the metabolic consequences of altering such an important metabolic gene. Although inhibitors of mutant IDH1 have been discovered, their lack of success in the clinic has led to the belief that alternative pathways could serve better as potential vulnerabilities [15]. One proposed vulnerability of IDH1*^mut^* cancer cells is the depletion of reduced nicotinamide adenine dinucleotide NADH, which triggers autophagy and cytotoxicity selectively for IDH1*^mut^* gliomas [16]. Accordingly, an inhibitor of NAD^+^ salvage pathway has been tested for lower grade gliomas with the goal to be added to clinical trials [17]. The metabolic consequences of depleting NAD^+^ were not fully explored. Although the reported mechanisms by which depletion of NAD^+^ leads to cell death was found to be autophagy; however, these investigations left a lot of unanswered questions related to the metabolic consequences of reducing such an important cofactor.

Herein, we used Raman imaging microscopy to decipher the mechanism of action of FK866, an inhibitor of NAD^+^ salvage pathway, in vitro and in vivo. While the cells seem to be sensitive to the drug by displaying a decrease in viability and decreased lipid and protein content, however, in vivo a compensatory mechanism that allows the cells to overcome the loss of lipids and sustain tumor growth could be present. This is clear by the increase in lipid content in tissue from mice treated with FK866. Using significant features or contributions of the drug in the Raman spectra, we can track the FK866 in tissue and map its distribution or the distribution of its changes. Therefore, we show that Raman imaging microscopy can report on global metabolic changes in live cells and tissue and can be used for the tracing of drugs or drug-induced local changes.

## 2. Materials and Methods

### 2.1. Raman Imaging Microscopy

Samples used for Raman spectroscopy were transferred into 35 mm sterile glass bottom dishes (Ibidi) and cultured for 72 h in the presence and absence of 12.5 nM FK866. Raman spectra were acquired using a DXR2xi Raman microscope (ThermoFisher Scientific, Madison, WI, USA) with 9.9 mW of 532 nm laser at the sample through a 60X water immersed confocal objective (N.A. = 1, LUMPLFLN60XW Olympus America Inc., Waltham, MA, USA) at 0.5 s exposure time for a 1 μm pixel size, for the 50 to 3200 cm^−1^ spectral region. Spectra from the non-nucleus areas of 10 × 10 pixels of at least three cells were collected and subsequently background-corrected using the Raman silent region. Chemical images were produced by the peak area function of the assigned peaks using Thermo Fisher Scientific OMNICxi software (ThermoFisher Scientific™, Madison, WI, USA). For tissue experiments, unstained quartz slides of 5 µm tissue from mice brain (vehicle or treated) were obtained from Histoserv., Inc. (Germantown, MD, USA). The Raman acquisition was done under the same general conditions but using a 100X air/dry objective (N.A. = 0.9, MPLN100X, Olympus America Inc., Waltham, MA, USA).

### 2.2. Cell Culture and Viability Measurements

HT1080 were maintained in Dulbecco minimal essential medium (DMEM) supplemented with 10% FBS (Invitrogen, Carlsbad, CA, USA) and Pen/Strep. Cells were plated at 2 × 10^6^ per cell culture dish with 12.5 nM FK866 dissolved in DMSO or an equivalent volume DMSO. After 72 h, cells were quenched and collected as follows. Media was aspirated from adherent cells, washed with PBS, and then 1 mL of ice-cold water was added to the flask and immediately placed onto dry ice. These frozen cell layers were then scraped and transferred to a 15 mL tube and placed into dry ice until metabolites extraction. For cell viability, 0.5 million cells were incubated with FK866 for 72 h. Following treatment, cells were trypsinzed and counted by mixing with Trypan Blue and using a Vi-CELL XR (Beckman Coulter Inc., Atlanta, GA, USA) cell viability analyzer.

### 2.3. Sample Preparation for Nuclear Magneti Resonance (NMR) Spectroscopy

Cell suspensions in water were lysed via three cycles of freeze-thawing, including a 5 min sonication process in an ice-water bath after samples were thawed. Fifty microliters of this cell lysate were put aside for further protein quantification using the Bicinchoninic Acid (BCA) Protein Assay Kit (Thermo Fisher Scientific). Subsequently, 2 mL of ice-cold methanol were added to the cell lysates and incubated in ice with agitation for 10 min. Two milliliters of ice-cold chloroform were then added and also incubated in ice with agitation for 10 min. Then, samples were centrifuged at 12,000 rpm for 20 min at 4 °C. Bottom (lipophilic) layer was transferred to a glass vial and dried under a gentle stream of N2. The sediment was reconstituted in 180 µL of CDCl3 (0.03% v/v tetramethylsilane (TMS):CD3OD (2:1) and transferred to a 3 mm-NMR tube. 

### 2.4. ^1^H NMR Spectral Acquisition and Analysis

Single-pulse ^1^H NMR spectra were acquired on a Bruker Avance III 600 MHz spectrometer (Structural Biophysics Laboratory, NCI, Frederick, MD, USA) operating at a probe temperature of 298 K. Pulse sequence employed was zgpr (TopSpin 3.5, Bruker Biospin, Billerica, MA, USA) for suppression of the residual water resonance. For each spectrum, 64 scans were acquired, with a relaxation delay of 3 s, a spectral width of 10,800 Hz, and a time domain of 32 K points. Spectra were referenced to the TMS internal standard signal (s, δ = 0.00 ppm), zero-filled to 64 K points, phased, and baseline-corrected using an ACD Labs Spectrus Processor (2016) (Advanced Chemistry Development Inc., Ontario, Canada), and an exponential line broadening function of 0.30 Hz was applied. Resonances arising from lipids were normalized to the TMS resonance peak and to the protein content.

### 2.5. Lipidomics Using Ultra Performance Liquid Chromatography and Quadrupole Time-of-Flight Mass Spectrometry (UPLC-QTOF-MS)

HT1080 cells were grown in DMEM supplemented with 5% FBS and treated with 12.7 nM FK866 drug or DMSO as control. After 72 h of incubation, the media was removed, and cells were washed with cold PBS. Cells were scrapped on dry-ice and the cell lysate was subjected to freeze though and sonication for subsequent metabolite extraction. Cell samples were centrifuged at 400 rpm for 10 min and the supernatant was discarded. Resulting pellets were quenched in 0.6 µL MilliQ water, centrifuged at 400 rpm for 5 min to discard the supernatant, and snap-frozen on dry ice prior to storage at −80 °C. Each cell pellet was placed on ice, resuspended in 450 µL of chilled MilliQ water, and then lysed for 30 s with sonicator probe set at 20 A. Samples were placed on dry ice for 3 min and returned to a sonicator ice bath for 10 min. A total of 10% of the resulting lysate was collected to perform Bradford protein quantification. The Bligh and Dyer biphasic liquid extraction was performed on remaining lysates using a ratio of 2:2:1.5 chloroform/water/methanol [18]. The chilled aqueous reagent was spiked with internal standard (nitrodracylic acid and isocaramidine sulfate), added to the extract solution, vortexed, and incubated on ice for 10 min. The chilled chloroform reagent was added to the extract solution and placed on rotator for 60 min on ice. The samples were centrifuged at 12,000 rpm for 20 min at 4 °C. The resulting two phases (upper aqueous polar and lower organic lipid) were separated and the remaining protein disk layer was discarded. As described by Altadill et al., 1:1 acetonitrile was added to each phase prior to centrifugation and concentration under rotary evaporator, snap freezing with dry ice, and storing at −80 °C. Prior to liquid chromatography-mass spectrometry (LC-MS)lipidome analysis, the organic and polar phase were reconstituted and combined in a buffer containing methanol: acetonitrile (ACN): water as described previously [19]. Pooled quality control (QC) samples were prepared by combining 10% of each sample. Lipidomic LCMS analysis was performed on each sample using an Acquity UPLC CSH 1.7 µm, 2.1 × 100 mm column (Waters Corp., Milford, MA, USA) as described previously [19].

Mass spectrometry data analysis was performed following the logical binning of mass features using the XCMS software package in R Studio (Boston, MA, USA) as described previously [19]. LIPID MAPS Lipidomics Gateway and Human Metabolome Database queries were used to assign putative identities to mass features using based on mass accuracy within 0–5 mDa.

### 2.6. Mice Experiments

Intracranial orthotopic mouse models with an IDH1mut glioma cell line were established according to an approved animal study proposal NOB-008 by NCI-Animal Use and Care Committee (ACUC). Briefly, cells were harvested, washed with PBS, and counted. The resulting pellet was resuspended in Hank’s Balanced Salt Solution (HBSS) and 5 μL of the cell suspension were injected stereotactically into the striatum of female severe combined immunodeficiency (SCID) mice (6–8 weeks old, Charles River Frederick Research Model Facility) using a stereotactic device (coordinates, 2 mm anterior and 2 mm lateral from bregma, and 2.5 mm depth from the dura). Tumor growth was monitored for neurological symptoms daily. Mice were randomized into two groups (vehicle and treated; n = 10 and n = 11, respectively) and FK866 treatment (30 mg/kg, intra peritoneal, daily) started on day 68 via gavage and ended on day 82 (lasted for 14 days). When the animals reached end-points, they were euthanized via perfusion with 4% paraformaldehyde (PFA) under anesthesia and their brains were dissected for histopathological examination and immunochemistry analysis. For comparison of survival curves, a log-rank (Mantel–Cox) test has been used. Haematoxylin and eosin( H&E) stained, and unstained tissue slides were obtained from Histoserv. Inc. (Germantown, MD, USA). Adjacent slides were cut for Raman analysis, therefore each slide analyzed was confirmed to have tumor via histology.

## 3. Results

### 3.1. Raman Spectroscopy of Live HT1080 Cells Revealed Decreased Lipids upon FK866 Drug Treatment

**Figure 1:**
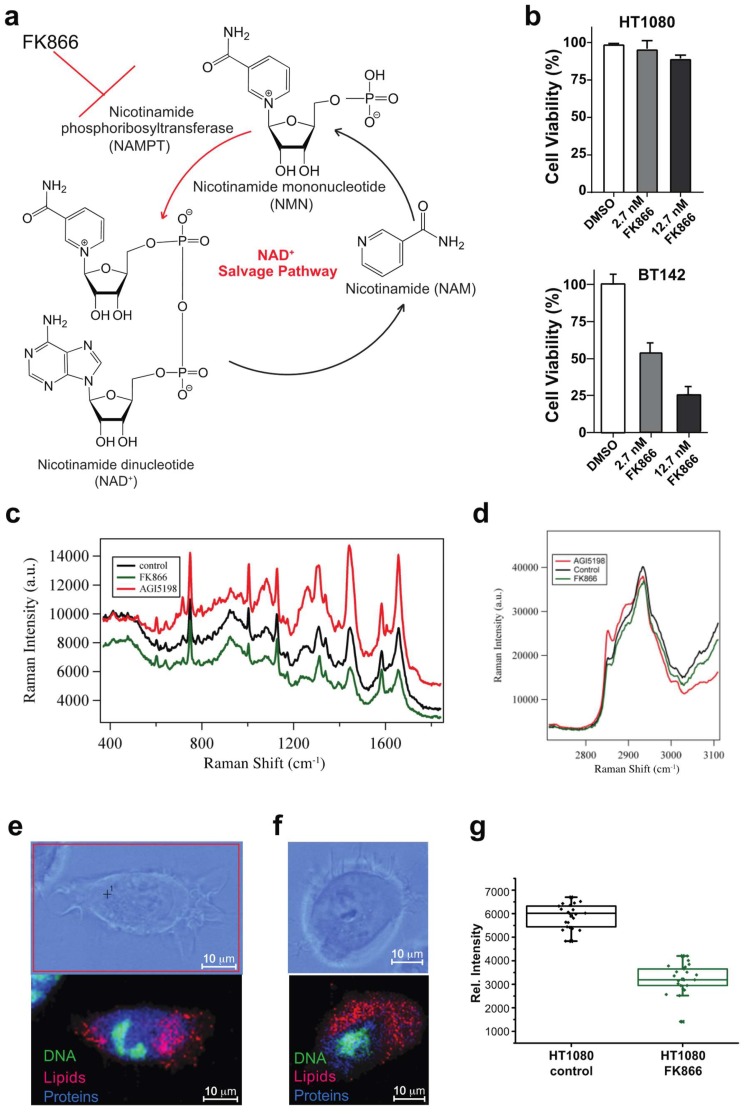
Detection of metabolic changes in live cells via Raman imaging microscopy. (**a**) FK866 inhibits the salvage pathway of NAD^+^ biosynthesis. (**b**) Viability measurements of HT1080 and BT142 cells in presence of different concentrations of FK866. (**c**) Overlay of Raman spectra for HT1080 in presence of FK866 (green) and AGI-5198 (red). Averaged Raman spectra of cytoplasm (*n* = 3) over an area of 10 × 10 pixels are displayed. (**d**) Selected region of the Raman spectra to show the detection of changes in lipids and proteins as a response to either FK866 (green) or AGI-5198, inhibitor of IDH1 mutation (red) treatment of HT1080 cells. (**e**,**f**) Distribution of DNA (green), lipids (red) and proteins (blue) in the untreated HT1080 (**e**) or FK866 treated cells (**f**). (**g**) Box and whisker plots that reflects the distribution of intensities coming from the lipid peak (2845 cm^−1^) from *n* = 3 cells and selecting regions of the cells that exclude the nucleus to assess the variability of the data and the decreased in lipid content upon FK866 addition.

In this study, we tested whether Raman imaging microscopy is sensitive enough to detect changes at the single cell level as a function of metabolic drugs. FK866 inhibits the salvage pathway of NAD+ and was proposed to be very effective and selective in killing IDH1mut glioma (Figure 1a). AGI-5198 is a specific inhibitor of the IDH1 mutation and was used here as a control of IDH1mut-specific metabolic changes [20]. This inhibitor prevents the formation of D-2-hydroxyglutarate by binding to the enzyme without perturbing the activity of the wild type monomer. We tested the effect of these drugs on two cell lines: BT142 which was isolated from an oligoastrocytoma grade III brain tumor of a 38-year-old male and HT1080, which was isolated from a fibrosarcoma of a 35-year old male. Both cell lines were purchased from American Type Culture Collection (ATCC). Raman spectra shown in the Figure 1 were recorded in regions of the cells that were non-nuclear and represented the average of 100 spectra from an area of 10 × 10 pixels. Peak intensity was at position 2845 cm^−1^, which was assigned to lipids that were used as a proxy for lipid content. Assignments used for displaying distribution of nucleic acids, proteins, and lipids are shown in Table 1. The viability of BT142 cell lines dropped from 100% to 20% at the highest concentration of the drug, while HT1080 displayed a marginal effect (Figure 1b). By comparing the full range of Raman spectrum (50–3200 cm^−1^), we noticed marked differences in the lipid and protein specific bands (2845–2930 cm^−1^) (Figure 1d). Through mapping the intensity of proteins and lipids in the cells, we observed a marked difference in their distribution upon drug treatment (Figure 1e,f, and Table 1). When random regions were selected from the non-nucleus areas of the cells, we found that lipid content was markedly decreased upon treatment with FK866 in live cells (Figure 1g). To test whether this effect was due to the presence of IDH1 mutation, we added AGI-5198, and as expected, the lipid content was increased (Figure 1d). Since the inhibitor AGI5198 corrected the mutation by inhibiting the production of D-2HG, the metabolite produced by the mutant cells, seeing a reversal of effect in the lipid content was expected.

### 3.2. Mass Spectrometry and NMR Identify Classes of Lipids Altered by FK866 Drug Treatment

Since the lipid distribution was variable inside the cytoplasmic region of the cells as shown in the Figure 1e,f, we confirmed our results obtained via Raman imaging microscopy by treating cells with FK866 for 72 h, extracting the lipid content, and performing untargeted metabolomics using NMR and mass spectrometry-based assays. Both methods confirmed the loss of lipid content in HT1080 cells in the presence of the FK866 drug that decreased NAD^+^ availability. Since NAD^+^ was contributing significantly to lipids synthesis it was not surprising to find these results; however, the specific lipids altered and their contribution to cell viability could not be discerned via Raman imaging alone [21].

**Figure 2:**
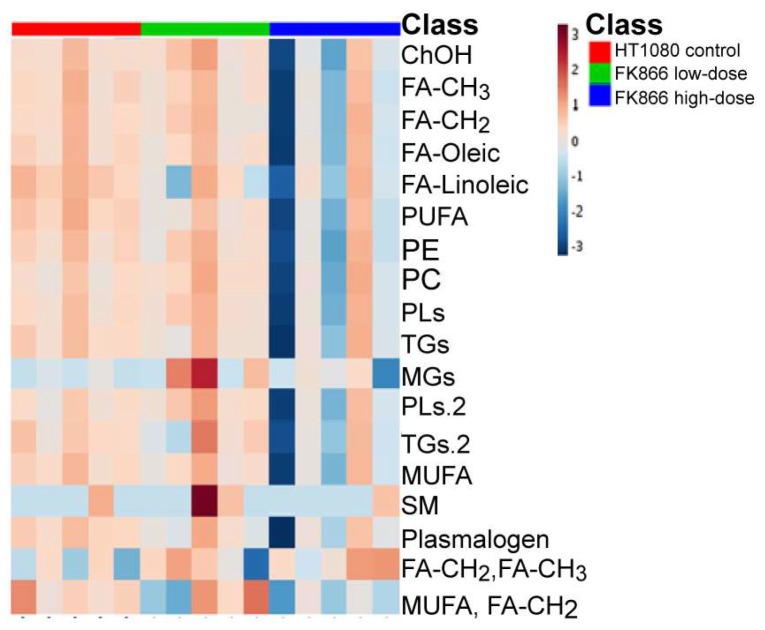
Heatmap of lipids from the NMR profiling of HT1080 cells (**red**), HT1080 cells treated with low dose of FK866 (**green**), or high dose of FK866 (**blue**). ChOH, cholesterol (unesterified); FA, fatty acid; PUFA, polyunsaturated fatty acid; PE, phosphatidylethanolamine; PC, phosphatidylcholine; PL, phospholipids; TGs, triglycerides; MGs, monoglycerides; MUFA, monounsaturated fatty acids; SM, sphingomyelin.

**Figure 3:**
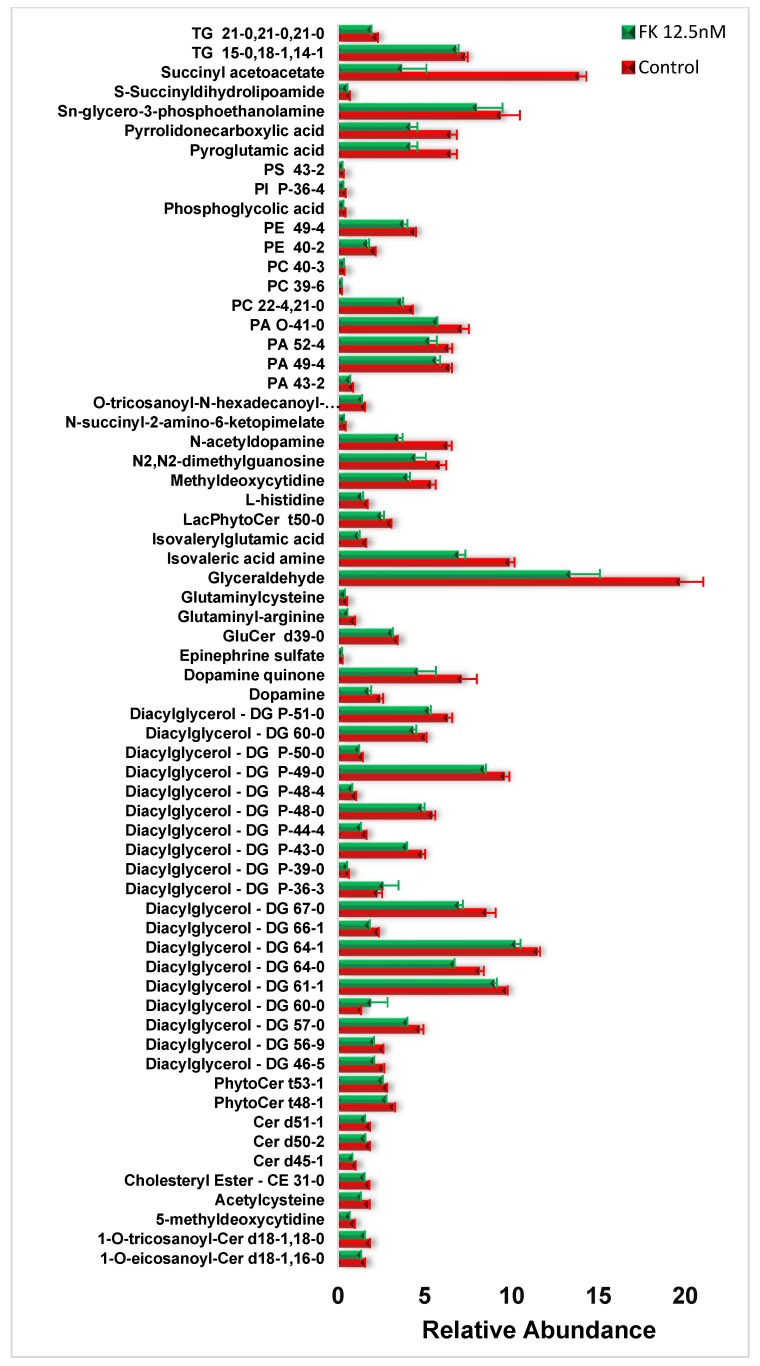
Mass spectrometry analysis of lipids in HT1080 cells treated with FK866. Metabolite changes upon treatment of HT1080 (**red**) with high dose of FK866 (**green**) obtained via LC/MS. CE, cholesteryl ester; FA, fatty acid; PE, phosphatidylethanolamine; PC, phosphatidylcholine; PS, phosphatidylserine; PA, phosphatyidic acid; PI, phosphoinositol; PIP, phosphoinositol monophosphates; PIP2, phosphoinositol bisphosphates; DG, diglyceride, TG, triglyceride; MG, monoglyceride; SM, sphingomyelin; Cer, ceramide; GluCer, glucosylceramide; LacCer, lactosylceramide; CAR, carnitine; NAT, N-acyltaurine.

**Figure 4:**
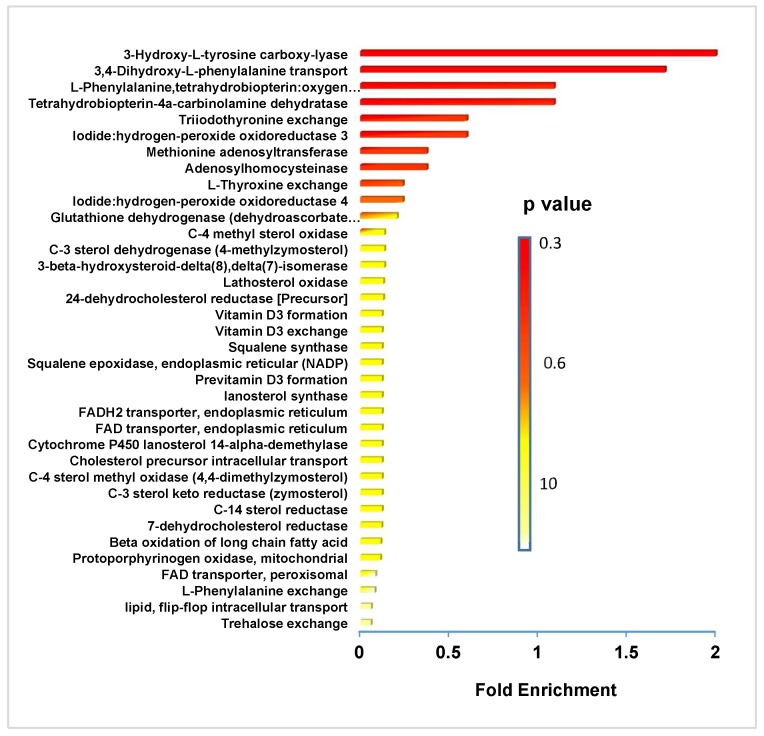
Metabolite sets enrichment overview for predicted enzymes and pathways associated with them. Metabolite set enrichment and pathway overview resulted from the mass spectrometry-based profiling of cells in response to the FK866 drug. The majority of metabolites were downregulated; therefore, the shown pathways are downregulated as well.

Using NMR, we observed downregulation of phosphatidylcholines (PCs) phosphatidylethanolamine (PEs), phospholipids (PLs), monounsaturated fatty acids (MUFAs), triglycerides (TGs), monoglycerides as well as sphingomyelin (SM), and plasmalogen (Figure 2). In the analysis done via LC/MS, we observed the vast majority of metabolites downregulated upon FK866 treatment, which comprised of diglycerides followed by triglycerides and ceramides (Figure 3). We then perform a metabolic enrichment analysis in order to correlate our findings with the enzymes that may be potentially involved in the response to treatment. Interestingly, the most significant changes were mapped to the enzymes that are part of the catecholamine pathway (Figure 4, Supplemental Figure S1, and Supplemental Table S1). Diglycerides, triglycerides, and ceramides are decreased as well as the common phospholipids. In addition, other metabolites are observed in the mass spectrometry analysis. With the exception for glutathione, which appeared to be upregulated upon treatment with FK866, the rest of the metabolites were downregulated. The following pathways were affected: lysine, tyrosine, glutamine and glutamate metabolism, glycerolipid, glycerophospholipid and sphingolipid metabolism, amino-acyl tRNA degradation, and the citric acid (TCA) cycle. The action of FK866 to decrease NADH content in cells was also confirmed via mass spectrometry analysis (Figure S2).

### 3.3. Raman Imaging Microscopy Reveals Increased Lipids in Tissue

**Figure 5:**
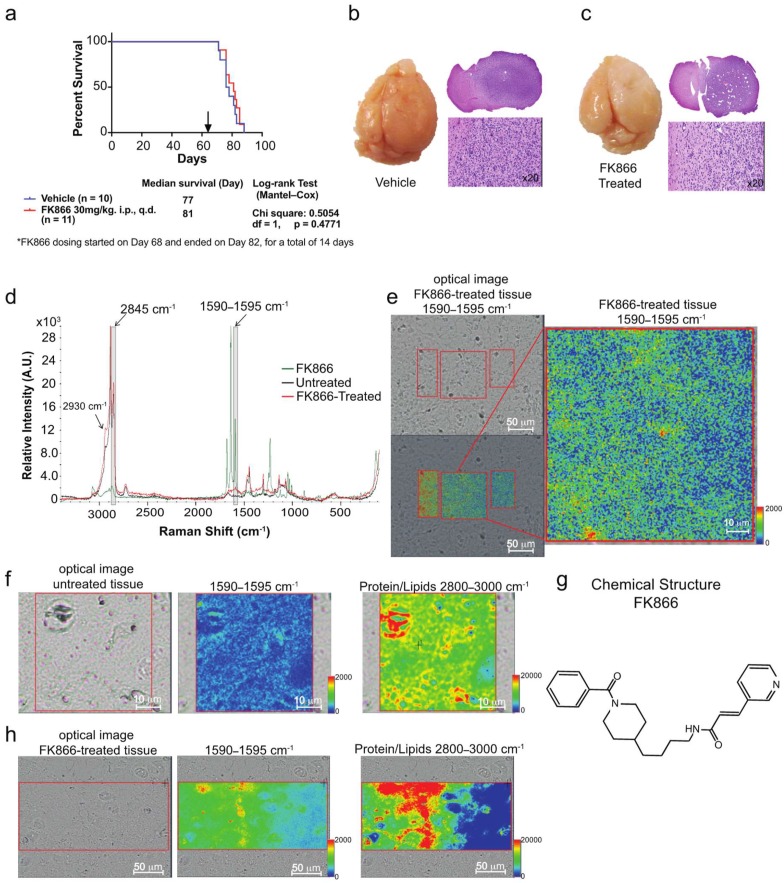
Detection of FK866-induced changes in the tissue of mice treated with FK866. (**a**) Mice survival after they were implanted with BT142 cells intracranially and were treated with FK866 (30 mg/kg). (**b**,**c**) Pictures of the whole brain and H&E staining for vehicle (**b**) and FK866 treated mice (**c**). (**d**) Overlay of the Raman spectra of tissue from the untreated (black), FK866 treated (red), and standard FK866 powder (green). Shaded grey areas are highlighting the peaks coming from either the FK866 drug (1590–1595 cm^−1^) or lipids (2845 cm^−1^) that were chosen for further mapping. (**e**) Mapping of FK866-induced changes in the treated tissue slides to show the distribution of drug in the tissue based upon the peak intensity at 1590–1595 cm^−1^. (**f**) Mapping of FK866-induced changes in untreated tissue slides (middle panel) or of proteins and lipids (right panel) to highlight the lack of intensity when attempting to map the intensity of 1590–1595 cm^−1^ (blue color), while the proteins and lipid frequencies display intensity. (**g**) Chemical structure of FK866. (**h**) Mapping of FK866-induced changes in treated-tissue slides (middle panel) or of proteins and lipids (right panel) to highlight the spatial co-localization of the drug or drug-induced changes in the areas of high cellularity.

To test the efficacy of FK866 in vivo, we designed an animal experiment in which we injected 0.5 million BT142 glioma cells intracranially in SCID mice, randomized the mice into two groups and start treatment after 68 days. Treating mice with a dose of 30 mg/kg for 14 days did not improve the survival of mice (Figure 5a), although we did notice a slight decrease in the cells infiltrating the surrounding upon drug treatment (Figure 5b,c). To understand the lack of efficacy in vivo, we then used Raman imaging microscopy to scan representative tissue from the two groups. Surprisingly, we found an increase in the lipid content in the treated mice group (Figure 5d). The increase in lipid content in the tissue from mice that were treated with FK866 could potentially be due to the brain microenvironment. The tumor microenvironment was very different than that of the cell culture and it has been shown to play a very important role in tumor maintenance and progression [22]. In fact, the brain was very rich in lipids and therefore a wealth of lipids was available for tumor cells to uptake while the tumor cells in culture were exposed to small quantities (µM) of lipids. The cell culture was homogenous while the brain microenvironment contained other types of cells as well as vessels that could support the growth and survival.

Moreover, we tested whether we could detect the distribution of the drug in tissue. Comparing the Raman spectrum of the pure drug with both treated and untreated samples, we found a Raman peak at 1595 cm^−1^ that was increased in the FK866-treated mice tissue and an overlap with a resonance from the FK866 drug, which we tentatively assigned to the Raman frequency coming from C=C of the drug [23]. Although, in theory, the tissue could contribute to this peak as well, in practice there was very little contribution from the tissue to the intensity at 1595 cm^−1^ in the absence of the FK866 drug. However, in the treated tissue, we could not distinguish the contribution drug-induced tissue changes that would lead to increases in the peak intensity at 1595 cm^−1^ and the signal coming from the drug itself. The intensity changes at 1595 cm^−1^ could have been due to the presence of FK866, or due to FK866-induced tissue changes. By accounting for the local effect of such a drug, one could at least indirectly map the presence of FK866. Therefore, using the 1595 cm^−1^ peak’s intensity, we looked at the distribution of FK866 in tissue (Figure 5e,h). Interestingly, the FK866 map shows a gradual spatial distribution, similar to diffusion-like, from an area displaying high drug concentration towards areas where the drug was not detected in the Raman spectrum (blue intensity regions). By comparing both the protein/lipid distribution and the FK866 distribution in the untreated tissue versus the FK866 treated ones (Figure 5f,h, respectively), we can clearly observe that the intensity assigned tentatively to the drug clusters in areas with a high content of protein and lipids is an indication of either the presence of the drug or drug-induced changes in tumor cells. The Raman image of the untreated tissue has a blue intensity, suggesting there was no presence of the drug or drug-induced changes (Figure 5f, middle panel). However, the corresponding image from the treated tissue clearly shows spots of red (high intensity) that overlap with the red spots coming from high protein and lipid content. This reveals that the drug was reaching the tumor cells or that the drug treatment led to very specific local changes in the tumor.

## 4. Discussion

### 4.1. Live Cell Imaging via Raman Imaging Microscopy

Raman imaging microscopy has become more prevalent for the use of biological samples, indicating that its potential for these kinds of applications will increase exponentially in the future. Herein, we evaluated whether the technique is sensitive enough to detect changes due to a metabolic drug treatment. We found that slight changes in peak intensities in Raman spectra for live cells due to treatment were reproducible (Figure 1g) and we validated them using two other techniques (LC/MS and NMR). This is of interest since peak intensities directly report on concentrations or the composition of the cell. We noticed a variable distribution of lipids in the cytoplasm, an observation that will be further investigated in detail in future studies. However, this study was not intended to address the quantification of cellular content and is more qualitative. We addressed the quantification of metabolic content in the cells using a more rigorous approach in a separate study [24].

### 4.2. Link between NAD^+^ Depletion and Decreased Lipid and Catecholamine Synthesis

FK866 is as an inhibitor of the NAD^+^ salvage pathway, and therefore NAD^+^ levels were diminished inside cells in presence of this drug. To date, most reports cite just two classes of enzymes to be affected by the depletion of NAD^+^ levels, since they utilize NAD^+^ primarily as a cofactor with low *K*_m_: poly (ADP-ribose) polymerase (PARP) and sirtuin (SIRT) enzymes. PARP enzymes are involved in DNA repair, gene expression, genomic stability and cell cycle amongst other processes [25,26], while SIRT enzymes play a major role in sensing the changes in the redox state of the cell resulting from either metabolic or other factors and in the stress response mechanism of the cell. Sirtuins also play a role in the chromatin regulation, and the development and differentiation of the cell [27,28]. However, NAD^+^-dependent enzymes are more diverse than the abovementioned classes and are involved in many biological processes from DNA damage repair to gene expression and calcium signaling [29]. Herein, we show that lipid content is decreased significantly in the presence of FK866, which suggests that the most likely enzymes involved in synthesis of lipids were also using NAD^+^. An enzyme that uses NAD^+^ as a cofactor is pyruvate dehydrogenase, the TCA cycle enzyme that performs pyruvate decarboxylation towards acetyl coenzyme A(acetyl-CoA). Since acetyl-CoA is the primary precursor of lipids synthesis, it is not surprising that a depletion in NAD^+^ would lead to inhibition of this enzyme and decreased lipids synthesis. However, additional experiments need to be performed to validate the inhibition of this enzyme.

Using NMR and mass spectrometry we validated the decrease in lipids observed via Raman imaging microscopy. In addition, we examined the classes of lipids that are altered the most and rationalized their contribution to cell growth and proliferation. Few classes of lipids were strikingly decreased in cells treated with FK866. Amongst them is arachidyl oleate, which is a long-chain fatty alcohol which is primarily located in the membrane, and its decrease would lead to a decrease in membrane and therefore cell division. PCs, PEs, and PLs are all decreased in both NMR and mass spectrometry experiments suggesting decreased membrane integrity and cellular division [30]. Catecholamine synthesis pathway involves phenylalanine and tyrosine; however, the enzymes involved in this pathway use tetrahydro-biopterin as a cofactor and its recycling is reliant upon the availability of NADPH. The depletion of NAD^+^ could affect NADPH pools leading to a reduction in the catecholamine synthesis pathway with consequences in proper neuronal function and signaling. Catecholamines are present in some regions of the brain where they control the activity of certain neurons via opening and closing of ion channels. Not surprisingly, defects in this pathway are linked with neurologic disorders [31]. Mice carrying mutations in the tyrosine hydroxylase, the rate-limiting enzyme of catecholamine biosynthesis, show a decrease in production of norepinephrine which leads to defects in long term memory formation [31].

### 4.3. Mapping of FK866 in Mice Tissue via Raman Imaging Microscopy

Tracing metabolites or chemicals has attracted major interest since the days of fludeoxyglucose positron emission tomography (^18^F-FDG PET) analysis for live animals or humans. The use of ^18^F-FDG PET in the clinic is a standard practice in any oncology field; however, it is limited to the tumors that consume large amounts of glucose and that are larger than 7 mm [32]. Herein, we tested whether we could track the fate of the FK866 drug in tissue after dissection from mice treated with this drug as proof of principle for the use or this techniques as a biosensor. Indeed, we could see a gradient-like pattern of drug distribution and clustering of this drug within areas of high protein and lipid content, another well-established marker for active proliferating cells. However, this is not without limitations. Firstly, the chemical structure of the drug has to contain unique regions of vibrations that do not overlap with those arising from the tissue vibrations under investigation. This could be alleviated by the addition of alkyne nitrile or deuterium adducts since this chemical bond is unique to the drug in question and vibrates in a Raman silent region [33]. Secondly, the sensitivity of the techniques is quite low; nevertheless, this could be improved via the use of surface-enhanced Raman scattering [34,35,36] (SERS) or via stimulated Raman [37]. We have explored the use of SERS, more specifically the use of gold nanoparticles to increase the signal to noise and an 10,000 increase in signal was obtained (unpublished data). Stimulated Raman has gained more momentum and few groups are specifically developing this method for the fast scanning of brain tissue [38,39,40]. It will be interesting to see the applicability of this method intra-operatively.

## 5. Conclusions

The use of Raman imaging microscopy for quantification of subtitle changes due to drug treatments is demonstrated. Herein, we identified and quantified changes in lipid content upon addition of the FK866 drug, which was not expected to decrease lipid content. The power of this imaging technique stands in the spatial distribution and the wealth of information that can be obtained from a single scan of a live cell. Therefore, in the future, this technique could be applied in determining the heterogeneity of the cells, or to identify tumor infiltrating cells located within the normal tissue. Other Raman-based methods could be developed to look at the synthesis of lipids, proteins and nucleic acids at the single cell level. Raman imaging microscopy has the potential to become the next single cell technology similar to single cell RNA-sequencing. The ability to obtain information at the single cell level could be exploited for the cases where material is sparse, such as immune cells. However, the limitations in mapping chemicals were highlighted as well and are related to the detection of low concentration of the drug and the identification of significantly altered peaks coming either from the drug without overlap from the sample or from drug-induced changes.

## Figures and Tables

**Table 1:** Raman peak assignment for tracing nucleic acids, proteins and lipids in live cells.

Band Position (cm^−1^)	Assignment		
785–788	Pyrimidine Bases	Ring Breathing	Nucleic Acids
1655–1662	Amide I	C=O Stretching Mode	Proteins
2850–2880	CH	Stretching	Lipids

## Supplementary Materials

The following are available online at http://www.mdpi.com/2079-6374/9/1/5/s1, Table S1: Fold change metabolites identified via mass spectrometry to be altered in the HT1080 cell treatment and the pathways. Figure S1: Heatmap of metabolites obtained from LC/MS from FK866 treated cells versus control. Figure S2: Quantitative changes in NADH as a function of FK866 treatment.
