# Supplementary material for: Detection of Metabolic Changes Induced via Drug Treatments in Live Cancer Cells and Tissue Using Raman Imaging Microscopy
Source: Biosensors (Basel). 2018 Dec 28;9(1):5. doi: 10.3390/bios9010005 (PMC6468689; doi:10.3390/bios9010005)
Supplement: Supplementary file 1 [file biosensors-09-00005-s001.pdf]

# Supplementary Materials

*Article*

# Detection of Metabolic Changes Induced via Drug Treatments in Live Cancer Cells and Tissue Using Raman Imaging Microscopy

**Mioara Larion [1], Tyrone Dowdy [1], Victor Ruiz-Rodado [1], Matthew W. Meyer [2], Hua Song [1], Wei Zhang [1], Dionne Davis [1], Mark R. Gilbert [1] and Adrian Lita [1,*]**

[1] Neuro-Oncology Branch, Center for Cancer Research, National Cancer Institute, National Institutes of Health, Bethesda, MD 20892, USA; mioara.larion@nih.gov (M.L.); tyrone.dowdy@nih.gov (T.D.); victor.ruizrodado@nih.gov (V.R.-R.); hua.song@nih.gov (H.S.); zhangwe@mail.nih.gov (W.Z.); ddavis913@icloud.com (D.D.); mark.gilbert@nih.gov (M.R.G.)

[2] 5225 Verona Road, ThermoFisher Scientific, Madison, WI 53711, USA; matt.meyer@thermofisher.com

* Correspondence: adrian.lita@nih.gov; Tel.: +1-240-760-7149

**Table S1.** Metabolites and pathway altered for HT1080 cells treated with FK866 from LC/MS.

| Compound ID | FC | log2(FC) | raw.pval | FDR.pval | pathway |
|---|---|---|---|---|---|
| Isovaleric acid amine | 0.70 | -0.51 | 0.0010 | 0.290 | Lysine metabolism |
| Diacylglycerol -DG 56-9 | 0.79 | -0.33 | 0.0017 | 0.290 | Glycerolipid metabolism |
| N-acetyldopamine | 0.55 | -0.87 | 0.0022 | 0.290 | Tyrosine metabolism |
| | | | | | |
| Diacylglycerol -DG 66-1 | 0.77 | -0.38 | 0.0051 | 0.290 | Glycerolipid metabolism |
| Pyroglutamic acid | 0.64 | -0.65 | 0.0052 | 0.290 | Glutathione metabolism |
| Pyrrolidonecarboxylic acid | 0.64 | -0.65 | 0.0052 | 0.290 | D-Glutamine and D-glutamate metabolism |
| Diacylglycerol -DG 64-1 | 0.89 | -0.17 | 0.0071 | 0.290 | Glycerolipid metabolism |
| Isovalerylglutamic acid | 0.70 | -0.52 | 0.0077 | 0.290 | Isovaleryl-CoA Dehydrogenase |
| Glyceraldehyde | 0.68 | -0.56 | 0.0089 | 0.290 | Glycerolipid metabolism |
| Diacylglycerol -DG 61-1 | 0.93 | -0.10 | 0.0090 | 0.290 | Glycerolipid metabolism |
| 1-O-eicosanoyl-Cer d18-1,16-0 | 0.85 | -0.23 | 0.0094 | 0.290 | Sphingolipid |
| PC 22-4,21-0 | 0.84 | -0.24 | 0.0101 | 0.290 | Glycerophospholipid metabolism |
| Cholesteryl ester - CE 31-0 | 0.83 | -0.27 | 0.0103 | 0.290 | Sterol Lipid metabolism |
| Phosphoglycolic acid | 0.66 | -0.61 | 0.0104 | 0.290 | Glyoxylate and dicarboxylate metabolism |
| Cer d45-1 | 0.82 | -0.28 | 0.0104 | 0.290 | Sphingolipid |
| Diacylglycerol 60-0 | 0.87 | -0.21 | 0.0117 | 0.290 | Glycerolipid metabolism |
| Diacylglycerol -DG P-44-4 | 0.80 | -0.32 | 0.0119 | 0.290 | Glycerolipid metabolism |
| Cer d51-1 | 0.84 | -0.25 | 0.0125 | 0.290 | Sphingolipid |
| 1-O-tricosanoyl-Cer d18-1,18-0 | 0.85 | -0.23 | 0.0125 | 0.290 | Sphingolipid |
| Methyldeoxycytidine | 0.74 | -0.43 | 0.0132 | 0.290 | DNA methyltransferase |
| Diacylglycerol -DG 64-0 | 0.82 | -0.29 | 0.0147 | 0.290 | Glycerolipid metabolism |
| GluCer d39 -0 | 0.90 | -0.16 | 0.0152 | 0.290 | Sphingolipid |
| DeoxyCer m18-0,20-0 | 0.86 | -0.22 | 0.0160 | 0.290 | Sphingolipid |
| Phyto-Cer t48-1 | 0.86 | -0.22 | 0.0179 | 0.290 | Sphingolipid |
| O-tricosanoyl-N-hexadecanoyl-sphing-4-enine | 0.90 | -0.15 | 0.0181 | 0.290 | Sphingolipid |
| PE 40-2 | 0.79 | -0.34 | 0.0181 | 0.290 | Glycerophospholipid metabolism |
| Diacylglycerol -DG P-49-0 | 0.88 | -0.19 | 0.0187 | 0.290 | Glycerolipid metabolism |
| Deoxy-Cer m18-0,26-0 | 0.83 | -0.27 | 0.0200 | 0.290 | Sphingolipid |
| PA 49-4 | 0.88 | -0.19 | 0.0205 | 0.290 | Glycerophospholipid metabolism |
| Diacylglycerol P-51 -0 | 0.83 | -0.27 | 0.0226 | 0.290 | Glycerolipid metabolism |
| Diacylglycerol -DG P-43-0 | 0.82 | -0.29 | 0.0234 | 0.290 | Glycerolipid metabolism |

| | | | | | |
|---|---|---|---|---|---|
| Diacylglycerol -DG P-39-0 | 0.80 | -0.32 | 0.0242 | 0.290 | Glycerolipid metabolism |
| Phyto-Cer t53-1 | 0.90 | -0.15 | 0.0243 | 0.290 | Sphingolipid |
| Diacylglycerol -DG 46-5 | 0.80 | -0.31 | 0.0249 | 0.290 | Glycerolipid metabolism |
| PS 43-2 | 0.78 | -0.35 | 0.0253 | 0.290 | Aminoacyl-tRNA biosynthesis |
| Diacylglycerol -DG P-50-0 | 0.85 | -0.23 | 0.0258 | 0.290 | Glycerolipid metabolism |
| Cer d50-2 | 0.84 | -0.26 | 0.0284 | 0.290 | Sphingolipid |
| L-histidine | 0.78 | -0.35 | 0.0299 | 0.290 | Aminoacyl-tRNA biosynthesis |
| N-succinyl-2-amino-6-ketopimelate | 0.77 | -0.37 | 0.0300 | 0.290 | Lysine metabolism |
| PA 52-4 | 0.82 | -0.28 | 0.0302 | 0.290 | Glycerophospholipid metabolism |
| Dopamine | 0.72 | -0.47 | 0.0302 | 0.290 | Tyrosine metabolism |
| Triacylglycerol - TG 21-0,21-0,21-0 | 0.85 | -0.23 | 0.0303 | 0.290 | Glycerolipid metabolism |
| PhytoLacCer t50 -0 | 0.81 | -0.31 | 0.0308 | 0.290 | Sphingolipid |
| Diaceylglycerol P-36 -3 | 0.79 | -0.33 | 0.0308 | 0.290 | Glycerolipid metabolism |
| Diacylglycerol -DG P-48-0 | 0.89 | -0.17 | 0.0316 | 0.290 | Glycerolipid metabolism |
| PE 49-4 | 0.85 | -0.23 | 0.0325 | 0.290 | Glycerophospholipid metabolism |
| PI P-36-4 | 0.70 | -0.51 | 0.0337 | 0.290 | Glycerophospholipid metabolism |
| N2,n2-dimethylguanosine | 0.76 | -0.40 | 0.0342 | 0.290 | Amino-acyl tRNA Degradation |
| S-Succinyldihydrolipoamide | 0.68 | -0.57 | 0.0344 | 0.290 | Citrate cycle (TCA cycle) |
| Diacylglycerol -DG P-48-4 | 0.78 | -0.35 | 0.0346 | 0.290 | Glycerolipid metabolism |
| PC 40-3 | 0.87 | -0.20 | 0.0381 | 0.313 | Glycerophospholipid metabolism |
| PA 43-2 | 0.78 | -0.36 | 0.0395 | 0.319 | Glycerophospholipid metabolism |
| Triacylglycerol - TG 15-0,18-1,14-1 | 0.93 | -0.10 | 0.0418 | 0.323 | Glycerolipid metabolism |
| Diacylglycerol -DG 67-0 | 0.81 | -0.30 | 0.0425 | 0.323 | Glycerolipid metabolism |
| PA O-41-0 | 0.80 | -0.32 | 0.0436 | 0.323 | Glycerophospholipid metabolism |
| Glutaminyl-arginine | 0.58 | -0.78 | 0.0449 | 0.323 | Amino acid degradation or cell-signaling |
| Acetylcysteine | 0.77 | -0.38 | 0.0456 | 0.323 | Glutathione metabolism |
| Dopamine quinone | 0.64 | -0.64 | 0.0457 | 0.323 | Tyrosine metabolism |
| Epinephrine sulfate | 0.87 | -0.21 | 0.0461 | 0.323 | Tyrosine metabolism |
| PC 39-6 | 0.91 | -0.14 | 0.0463 | 0.323 | Glycerophospholipid metabolism |
| Diacylglycerol -DG 57-0 | 0.85 | -0.24 | 0.0468 | 0.323 | Glycerolipid metabolism |
| Glutamylcysteine | 1.23 | 0.30 | 0.0472 | 0.323 | Glutathione metabolism |

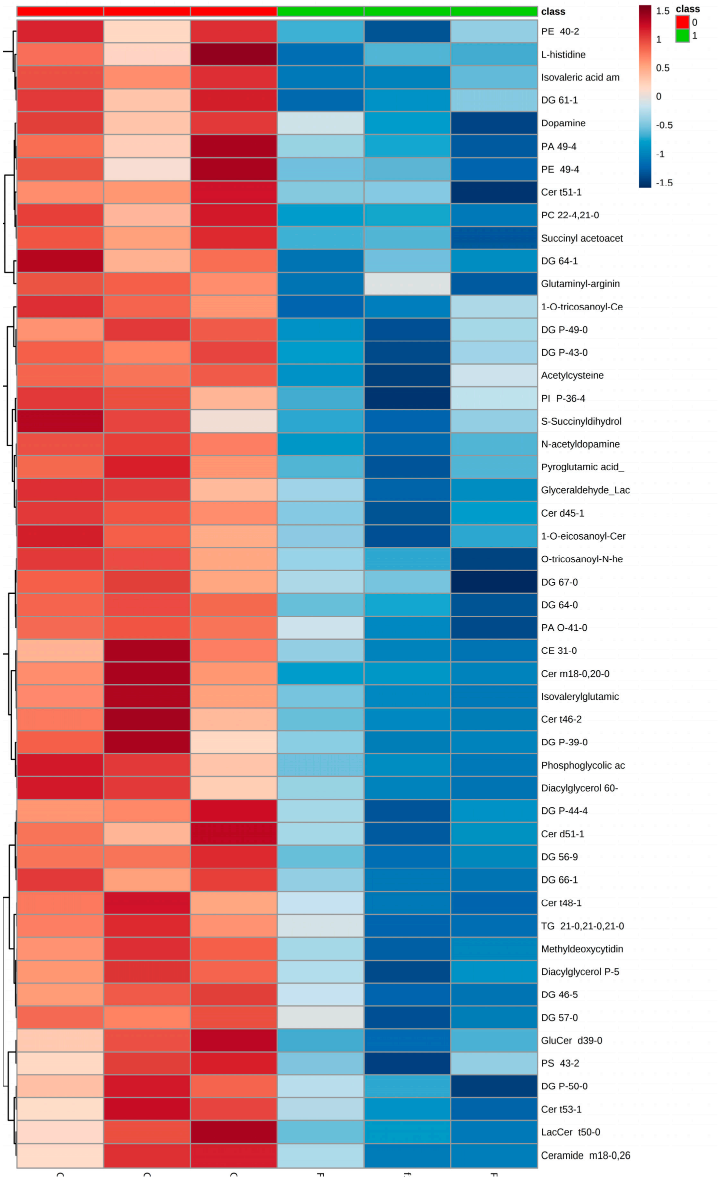


**Figure S1.** Heatmap of metabolite levels resulted as a function of FK866 treatment obtained via LC/MS. The following abbreviations were used: CE, Cholesteryl Ester, FA, Fatty Acid; PE, Phosphatidylethanolamine; PC, Phosphatidylcholine; PS, Phosphatidylserine; PA, Phosphatyidic Acid; PI, Phosphoinositol; PIP, Phosphoinositol monophosphates; PIP2, Phosphoinositol bisphosphates; DG, Diglyceride, TG, Triglyceride; MG, Monoglyceride; SM, Sphingomyelin; Cer, Ceramide; GluCer, Glucosylceramide; LacCer, Lactosylceramide; CAR, Carnitine; NAT, N-acyltaurine.

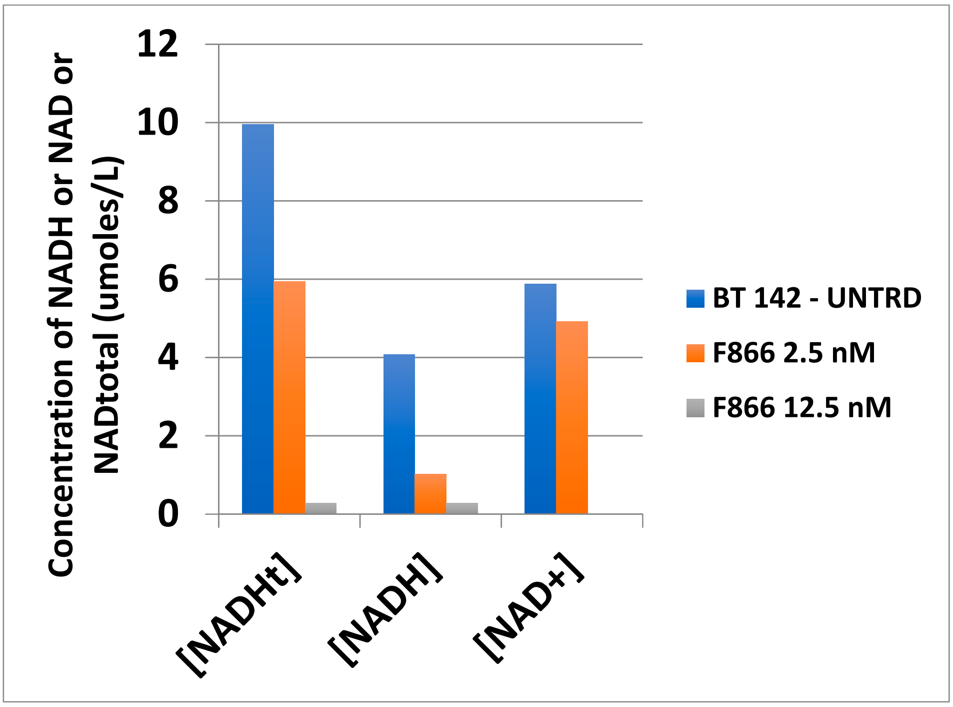


**Figure S2.** FK866 decreases the concentration of all NADH drastically in cells obtained via LC/MS. Total $NADH_t$ as well as the reduced and oxidized forms of NAD are decreased significantly in the cell after the treatment with FK866 (orange and grey bars). Levels of NADH in the untreated BT142 cell are shown in blue, low dose FK866 is shown in orange while high dose FK866 is shown in grey.
